# Berberine hydrochloride enhances innate immunity to protect against pathogen infection via p38 MAPK pathway

**DOI:** 10.3389/fimmu.2025.1536143

**Published:** 2025-02-28

**Authors:** Yi Xiao, Yingwen Cui, Yan Zhang, Wenqiao Fu, Yun Liu, Fang Liu

**Affiliations:** ^1^ Institute of Life Sciences, Zunyi Medical University, Zunyi, Guizhou, China; ^2^ College of Basic Medicine, Zunyi Medical University, Zunyi, Guizhou, China

**Keywords:** Berberine hydrochloride, innate immunity, p38 MAPK pathway, *Caenorhabditis elegans*, mouse

## Abstract

The p38 MAPK pathway, an evolutionarily conserved mechanism, plays a crucial role in defending hosts against bacterial infections in both mammals and nematodes. Activating p38 MAPK signaling has been identified as a promising strategy to strengthen innate immunity and enhance resistance to pathogenic infections across various organisms.Berberine hydrochloride (BH), an isoquinoline alkaloid derived from *Coptis*, is known for its diverse biological activities, including anticancer, antibacterial, anti-inflammatory, lipid-lowering, and hepatoprotective effects. However, its impact on innate immunity and the associated molecular mechanisms remains unclear. In this study, we discovered that 10 μM Berberine hydrochloride enhanced resistance against both Gram-negative pathogens, such as *Pseudomonas aeruginosa*, *Salmonella enterica* and Gram-positive pathogen *Listeria monocytogenes*. Notably, Berberine hydrochloride improved pathogen resistance by reducing bacterial load in the intestine. Screening of classical innate immune pathways in *Caenorhabditis elegans* revealed that Berberine hydrochloride conferred protection against infections through the p38 MAPK pathway, specifically by activating p38/PMK-1 signaling in the intestine to bolster innate immunity. Furthermore, Berberine hydrochloride also stimulated innate immunity in mice via the p38 MAPK pathway and significantly reduced bacterial load in the lungs. These findings indicate that Berberine hydrochloride may have therapeutic potential for protecting host from infectious diseases.

## Introduction

Currently, bacterial infections are the primary cause of infectious diseases. Although antibiotics are initially highly effective in treating these conditions, their usage is increasingly limited by challenges such as antibiotic resistance, toxicity, and residual contamination (1). Innate immunity serves as the primary defense mechanism against pathogen infections. Given the advantages of traditional Chinese herbs, such as minimal side effects and a reduced likelihood of inducing drug resistance, research efforts increasingly focus on discovering new compounds from these herbs to identify potential innate immunity activators that could enhance protection against bacterial infections (2). Berberine hydrochloride, a natural alkaloid derived from *Rhizomacoptidis*, has been widely utilized in Ayurvedic and traditional Chinese medicine for centuries. It is well-documented for its diverse therapeutic properties, including anticancer effects (3), antibacterial (4), anti-inflammatory (5) and anti-neurodegenerative (6). However, the underlying molecular mechanisms through which it enhances innate immunity remain largely unexplored.

The innate immune system, an evolutionarily conserved mechanism present from nematodes to mammals, serves as the first defense line against microbial infections (7, 8). The innate immune system is activated during pathogen invasion, triggering an antimicrobial response to combat the infection (7, 9–12). *Caenorhabditis elegans* has been used as tractable model to study host-bacterial interaction which reveals several signaling pathways that are involved in controlling innate immunity, such as the PMK-1/p38 MAPK pathway (13, 14), the DAF-2/DAF-16 pathway (15), the MPK-1/ERK MAPK pathway (16). The p38 MAPK pathway, a fundamental component of the innate immune response to pathogen infection, is evolutionarily conserved across species, from nematodes to mammals (10). In this pathway, the signaling cascade progresses from NSY-1/ASK1 (MAPK kinase kinase) to SEK-1/MKK3/MKK6 (MAPK kinase), and subsequently to PMK-1/p38 (MAPK) (13, 17, 18).

This study explored the role of Berberine hydrochloride in enhancing host defenses against pathogen infection. Pathway screening revealed that Berberine hydrochloride exerts its protective effects through the p38 MAPK pathway. Specifically, it increased resistance to bacterial infections by activating PMK-1/p38 MAPK in the intestine. Additionally, Berberine hydrochloride was found to enhance innate immunity in mice via activation of the p38 MAPK pathway. The evolutionary conservation of the p38 MAPK pathway suggests that Berberine hydrochloride-mediated innate immunity may be universally applicable across species, from nematodes to mammals.

## Results

### Berberine hydrochloride defenses against pathogen infection in *C. elegans*

**Figure 1:**
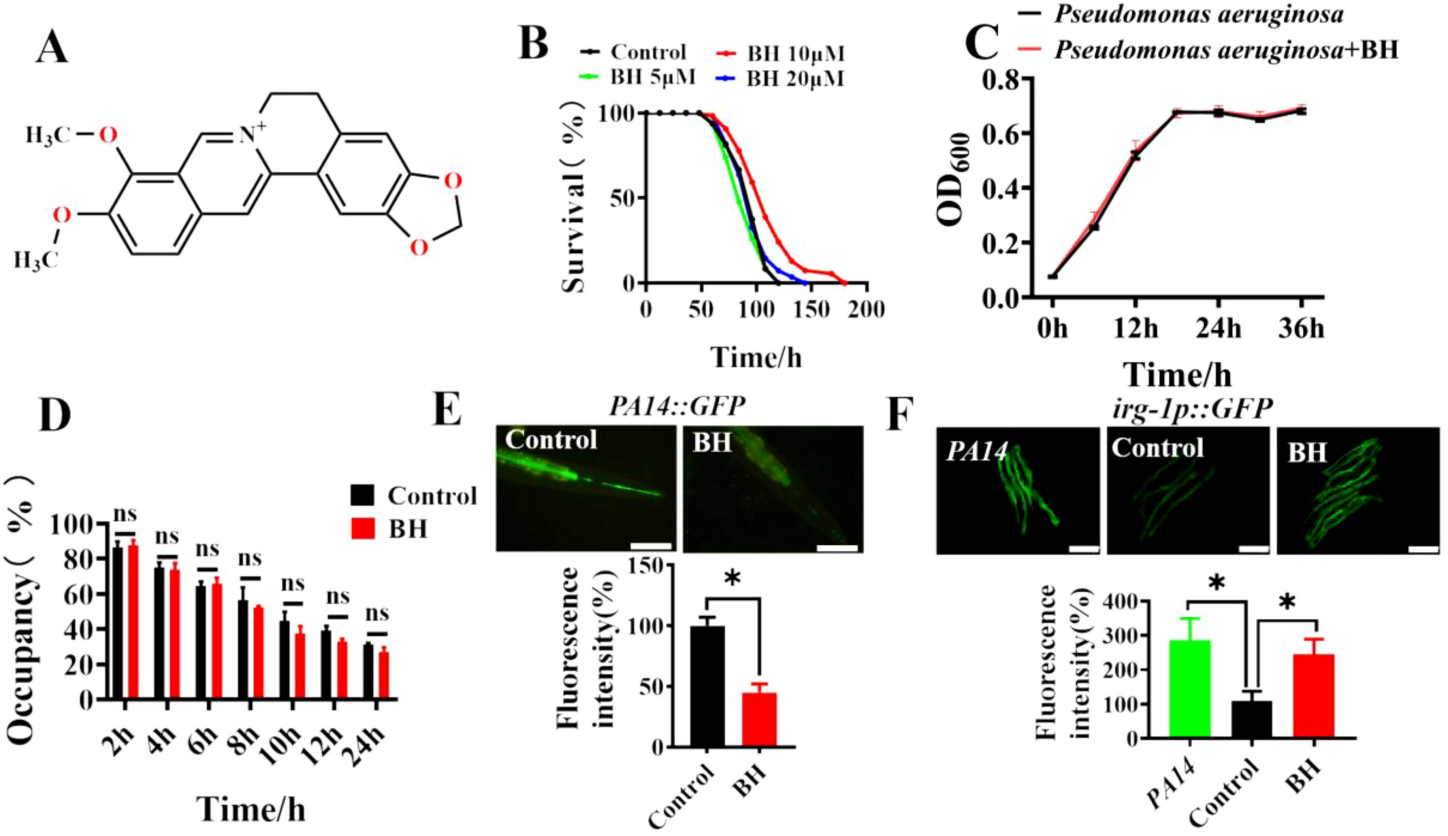
Berberine hydrochloride defenses against pathogen infections in *C. elegans*. **(A)** The chemical structure of Berberine hydrochloride (BH). **(B)** Survival of N2 hermaphrodite worms exposed to increasing concentrations of BH in response to *P. aeruginosa* PA14 infection. (*P < 0.05; log-rank test). (n> 40). See **Supplementary Table S1** for survival data. **(C)** BH (10 μM) did not inhibit the proliferation of *P. aeruginosa* PA14. **(D)** Animals were placed on a small spot of *P. aeruginosa* in a 3 cm plate and monitored over time for their presence or absence on the lawn after BH (10 μM) treatment. ns (no significance). Error bars represent mean ± SEM of 3 independent biological replicates. **(E)** WT animals-treated 10 μM BH were exposed to *P. aeruginosa* expressing GFP for 48 hours and then visualized using a Zeiss Axioskop 2 plus fluorescence microscope. Scale bars: 50 μm. (n≥ 10). These results are mean ± SEM of three independent experiments. (*P < 0.05, unpaired t-test). **(F)** The levels of *irg-1::GFP* after treatment 10 μM BH. (n≥ 20). Scale bars: 100 μm. (*P < 0.05, unpaired t-test). Error bars represent mean ± SEM of 3 independent biological replicates. Every independent experiment includes three biological replicates.

The chemical structure of Berberine hydrochloride (BH) was showed in **Figure 1A**. To test whether Berberine hydrochloride was able to increase pathogenic resistance, *C. elegans* were exposed to the human opportunistic pathogen *Pseudomonas aeruginosa* (PA14). Wild-type worms were treated with varying concentrations of Berberine hydrochloride (0 μM, 5 μM, 10 μM, 20 μM). The results indicated that 10 μM Berberine hydrochloride significantly improved survival to *P. aeruginosa* (**Figure 1B**; **Supplementary Table S1**). These findings suggested that Berberine hydrochloride enhances innate immunity in *C. elegans*. To determine whether this effect was due to inhibition of bacterial growth, a bacterial growth assay was conducted, showing that 10 μM Berberine hydrochloride did not suppress the proliferation of *P. aeruginosa* PA14 (**Figure 1C**). Moreover, our findings revealed that treatment with 10 μM Berberine hydrochloride has no effects on pathogen avoidance behavior in *C. elegans* (**Figure 1D**). Bacterial clearance is an essential aspect of host defense against pathogen infection (2, 19). To investigate whether Berberine hydrochloride affects bacterial accumulation, we examined its impact on *Pseudomonas aeruginosa* fusing with green fluorescent protein (GFP). Compared to control group, Berberine hydrochloride-treated worms showed a reduced accumulation of *P. aeruginosa*/GFP (**Figure 1E**). Furthermore, we assessed the effect of Berberine hydrochloride on the expression of the antimicrobial peptide gene *irg-1* (20), which was induced in exposure of *P. aeruginosa*. Our results revealed that Berberine hydrochloride increased the expression of *irg-1p::GFP* (**Figure 1F**). These findings indicate that Berberine hydrochloride may enhance pathogen resistance by lowering bacterial load in the *C. elegans* intestine.

### Berberine hydrochloride increases the resistance to pathogens

**Figure 2:**
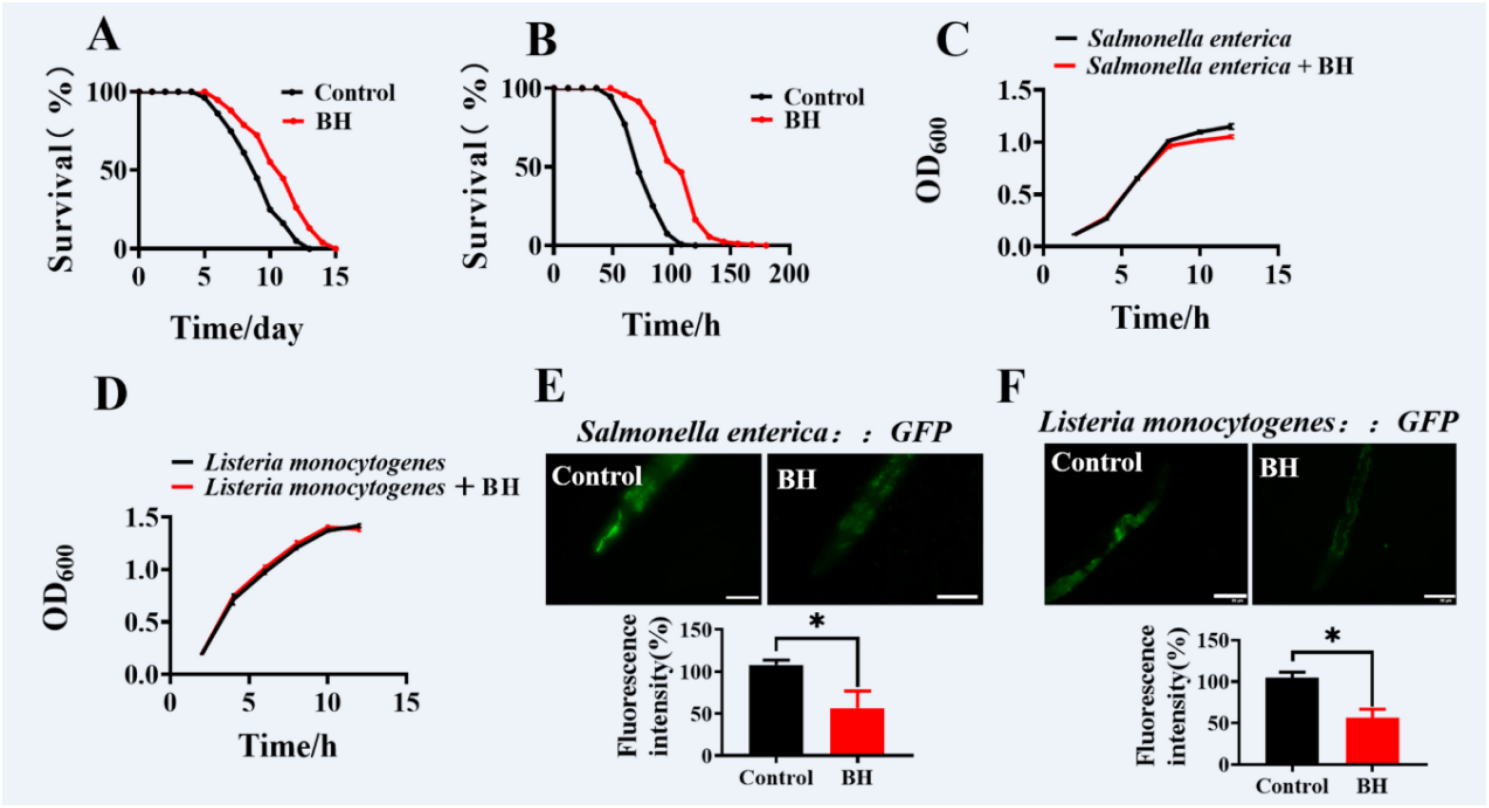
Berberine hydrochloride increases the resistance to pathogens. BH (10 μM) enhanced the resistance to *Salmonella enterica* **(A)**, and *Listeria monocytogenes* **(B)** in *C. elegans*. (*P < 0.05, log-rank test). (n> 40). See **Supplementary Table S1** for survival data. BH (10 μM) did not inhibit the proliferations of *Salmonella enterica* **(C)**, and *Listeria monocytogenes* **(D)**. **(E, F)** WT animals-treated 10 μM BH were exposed to *Salmonella enterica* **(E)** and *Listeria monocytogenes* **(F)** expressing GFP for 48 hours and then visualized using a Zeiss Axioskop 2 plus fluorescence microscope. Scale bars: 50 μm. (n ≥10). (*P < 0.05, unpaired t-test). Error bars represent mean ± SEM of 3 independent biological replicates. Every independent experiment includes three biological replicates.

To determine whether Berberine hydrochloride enhances resistance to other pathogens, we exposed worms to the Gram-negative bacterium *Salmonella enterica* and the Gram-positive bacterium *Listeria monocytogenes*. Supplementation with 10 μM Berberine hydrochloride significantly improved host survival (**Figures 2A,B**; **Supplementary Table S1**). To assess whether this effect was due to inhibition of bacterial growth, we conducted bacterial growth assays. The results demonstrated that 10 μM Berberine hydrochloride did not inhibit the growth of *S. enterica* or *L. monocytogenes* (**Figures 2C, D**). Since bacterial clearance is a critical aspect in host defense (2, 19). We next investigated whether Berberine hydrochloride affected bacterial accumulation. Compared to control animals, those treated with Berberine hydrochloride exhibited reduced accumulation of *S. enterica* and *L. monocytogenes* tagged with green fluorescent protein (GFP) (**Figures 2E, F**). These findings suggest that Berberine hydrochloride enhances resistance in both Gram positive and negative pathogens.

### Berberine hydrochloride promotes innate immunity through the p38 MAPK pathway

**Figure 3:**
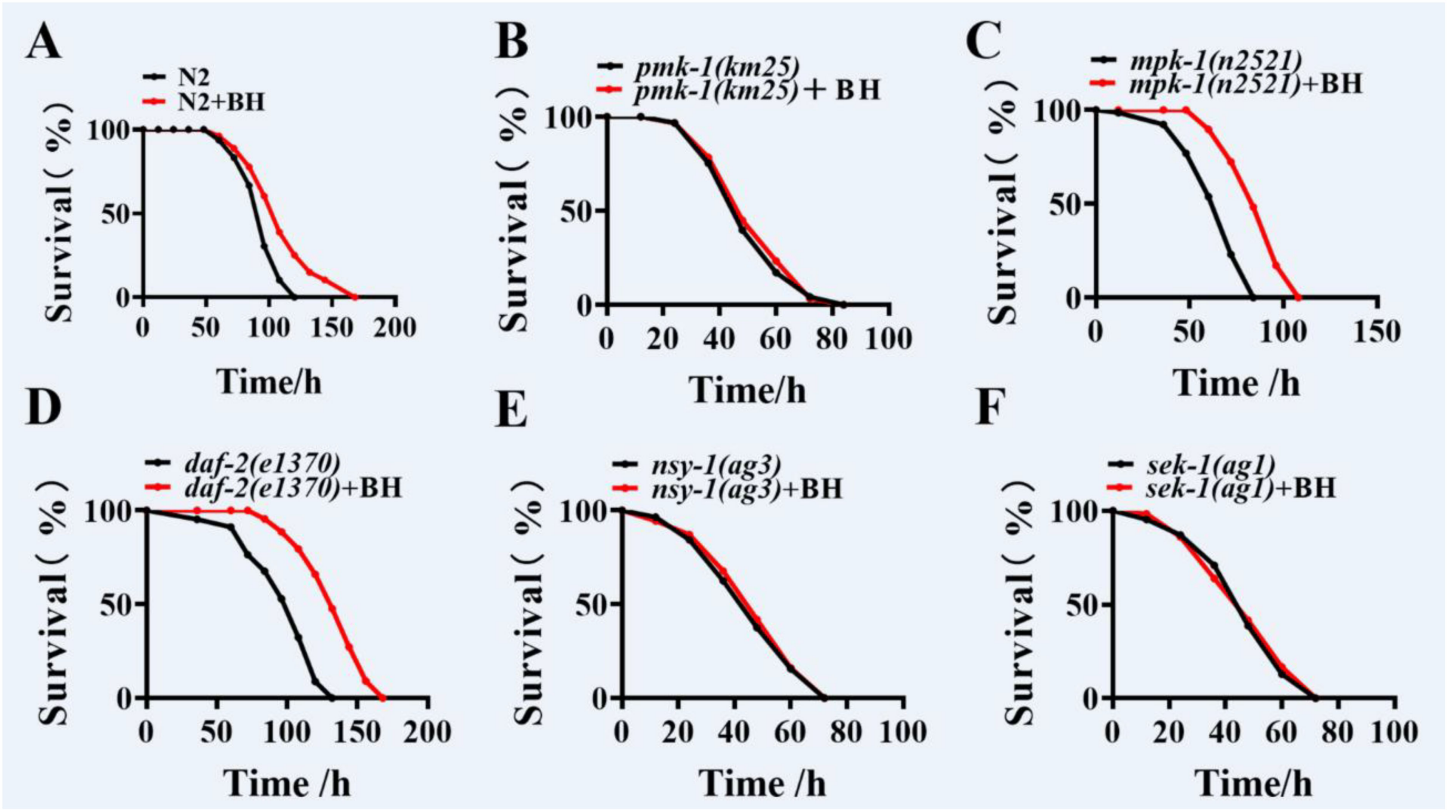
Berberine hydrochloride promotes innate immunity through the p38 MAPK pathway. **(A-F)** PMK-1/p38 MAPK was involved in BH-mediated innate immunity. BH enhanced resistance to *P. aeruginosa* PA14 in WT (N2) **(A)**, *daf-2(e1370)* **(C)**, *mpk-1(n2521)* **(D)**, but not in *pmk-1(km25)* mutants **(B)**. (log-rank test). (n> 40). See **Supplementary Table S1** for survival data. **(E, F)** Mutations in the components of the p38 MAPK pathway suppressed BH-mediated resistance of worms to *P. aeruginosa* PA14. **(E)** *nsy-1(ag3)*; **(F)** *sek-1(ag1)*. (n> 40). See **Supplementary Table S1** for survival data. The experimental berberine hydrochloride solution was added to the NGM culture plate. Every independent experiment includes three biological replicates.

To elucidate the molecular mechanisms underlying Berberine hydrochloride protective effects against pathogen infection, we examined several signaling pathways associated with innate immunity in *C. elegans*, including the p38 MAPK/PMK-1 pathway (13), DAF-2/DAF-16 insulin-like pathway (15), ERK MAPK/MPK-1 pathway (21). We found that Berberine hydrochloride failed to enhance resistance to *P. aeruginosa* PA14 infection in *pmk-1(km25)* mutants, compared to WT (N2) worms (**Figures 3A, B**; **Supplementary Table S1**). However, it significantly increased survival rates in *daf-2(e1370)* and *mpk-1(n2521)* mutants following *P. aeruginosa* PA14 infection (**Figures 3C, D**; **Supplementary Table S1**). We further investigated conserved signaling pathways, CRH-1/CREB, AAK-2/AMPK, and JNK-1/JNK, results revealed that Berberine hydrochloride enhanced survival in *crh-1(tz2)*, *aak-2(ok524)*, and *jnk-1(gk7)* mutants after infection, similar to its effects in wild-type worms (**Supplementary Figure S1A, D**). We then tested the core components of the p38 MAPK pathway, including the MAPK kinase NSY-1 and the MAPK kinase SEK-1. Berberine hydrochloride failed to confer resistance to *P. aeruginosa* PA14 infection in *nsy-1(ag3)* and *sek-1(ag1)* mutants (**Figures 3E, F**; **Supplementary Table S1**). These findings indicate that Berberine hydrochloride may enhance innate immunity in *C. elegans* primarily through the p38 MAPK pathway.

### Berberine hydrochloride activates p38 MAPK signaling in *C. elegan*s

**Figure 4:**
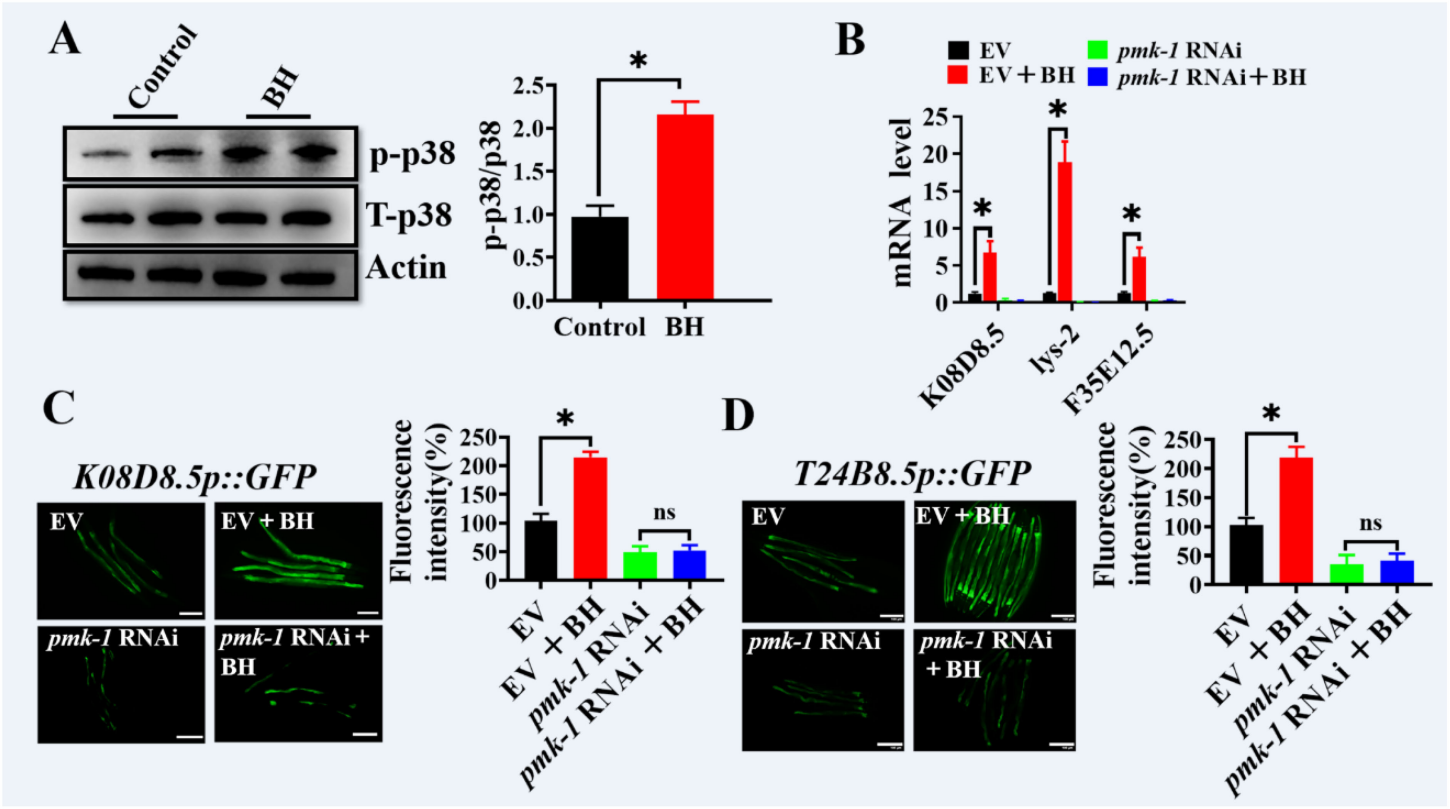
Berberine hydrochloride activates p38 MAPK signaling in *C. elegan*s. **(A)** The phosphorylation of p38 MAPK was elevated in WT worms (N2) exposed to BH. The right panel shows quantification of phosphorylated p38 MAPK levels. These results are mean ± SEM of three independent experiments performed in triplicate. (*P < 0.05, unpaired t-test). **(B)** The mRNA levels of three PMK-1/p38 MAPK targets *K08D8.5*, *lys-2* and *F35E12.5* in worms exposed to BH. These results are mean ± SEM of three independent experiments performed in triplicate. (*P < 0.05, unpaired t-test). **(C, D)** Expression of *K08D8.5p::GFP* **(C)** and *T24B8.5p::GFP* **(D)** were up-regulated in EV (empty vector) worms, but not in worms subjected to *pmk-1* RNAi worms, exposed to BH. The right panel shows quantification of fluorescence intensity. (n≥ 20). Scale bars: 100 μm. These results are mean ± SEM of three independent experiments performed in triplicate. (*P < 0.05, unpaired t-test). ns (no significance). Every independent experiment includes three biological replicates, and each independent Western Blot experiment included 2 biological replicates.

To determine whether Berberine hydrochloride activates the p38 MAPK pathway, we assessed the phosphorylation levels of PMK-1, a key marker of its activation (8, 22, 23). Our results showed that Berberine hydrochloride significantly increased the levels of phosphorylated PMK-1 protein in *C. elegans* (**Figure 4A**). We next examined the expression of PMK-1 downstream targeted genes, *K08D8.5*, *lys-2*, and *F35E12.5* (2, 24). Quantitative real-time PCR analysis revealed that these genes were up-regulated in Berberine hydrochloride-treated worms compared to control (**Figure 4B**). However, this up-regulation was abolished in *pmk-1* RNAi-treated worms (**Figure 4B**). Additionally, we evaluated the expression of *K08D8.5* and *T24B8.5* using transgenic worms expressing *K08D8.5p::GFP* and *T24B8.5p::GFP*. Higher GFP fluorescence levels were observed in Berberine hydrochloride-treated worms but were not detected in *pmk-1* RNAi worms (**Figures 4C, D**). These findings collectively demonstrate that Berberine hydrochloride may activate the p38 MAPK pathway in *C. elegans* after exposure to pathogens.

### Intestinal PMK-1 enhances resistance to pathogen infection after Berberine hydrochloride treatment

**Figure 5:**
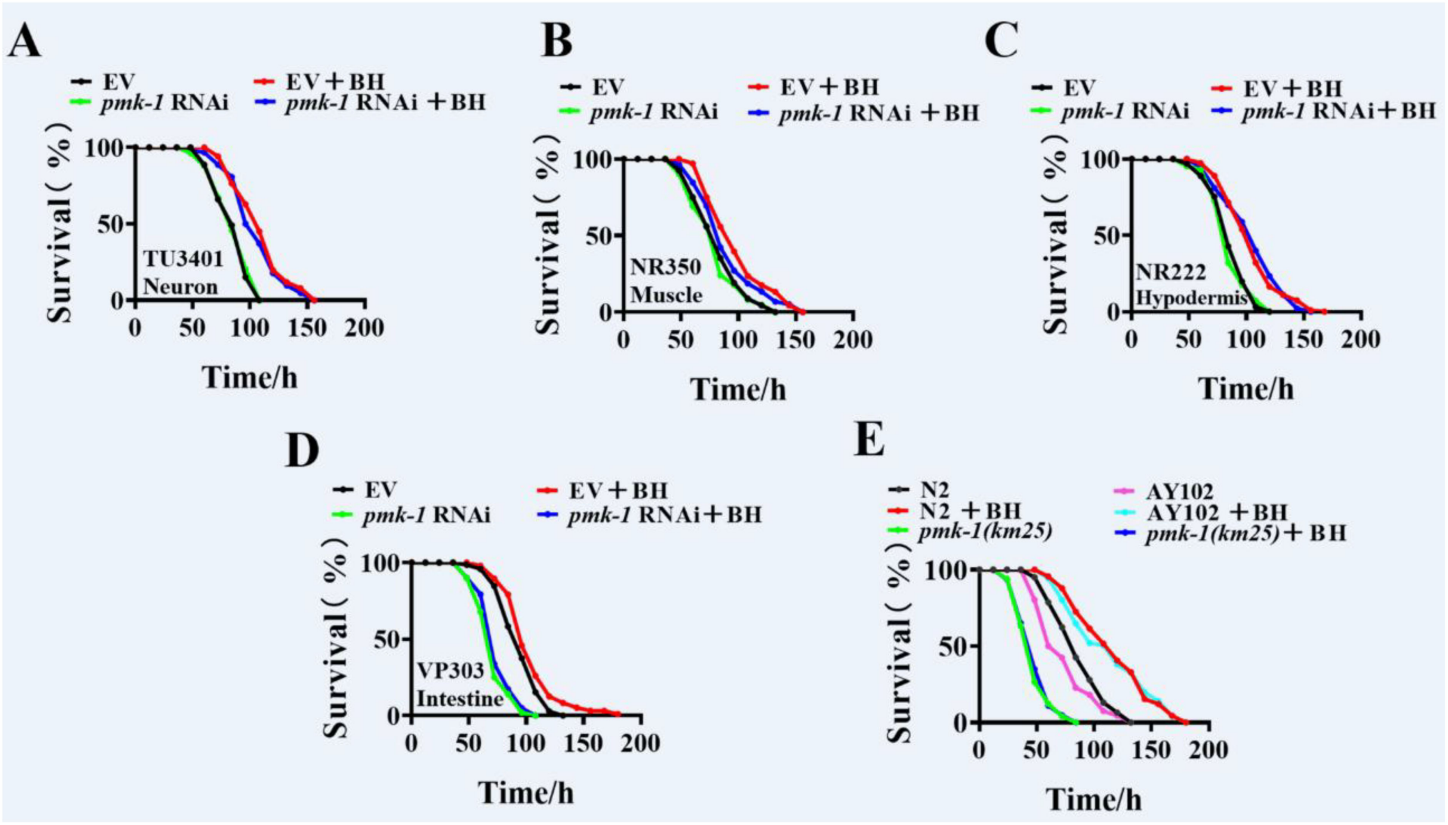
Intestinal PMK-1 enhances resistance to pathogen infection after Berberine hydrochloride treatment. **(D)** BH did not increase resistance to *P. aeruginosa* PA14 infection in intestinal-specific knockdown of *pmk-1*worms. However, RNAi of *pmk-1* in neuron **(A)**, muscle **(B)**, and hypodermis **(C)** respectively after BH treatment promoted the host survival during *P. aeruginosa* PA14 infection. **(E)** Expression of *pmk-1* under the intestinal-specific *vha-6* promoter (AY102) restored resistance against *P. aeruginosa* PA14 infection in *pmk-1(km25)* mutants after treatment with BH. (log-rank test). (n> 40). See **Supplementary Table S1** for survival data. Every independent experiment includes three biological replicates.

To evaluate the tissue-specific role of PMK-1 in response to *P. aeruginosa* infection following Berberine hydrochloride treatment, we conducted tissue-specific knockdown experiments using TU3401 (25, 26), NR350 (27), NR222 (27), and VP303 strains (28). Knockdown of *pmk-1* in neurons, muscle, or hypodermis after Berberine hydrochloride treatment enhanced host survival during *P. aeruginosa* PA14 infection (**Figures 5A–C**; **Supplementary Table S1**). However, RNAi-mediated knockdown of *pmk-1* in VP303 completely abolished the protective effect of Berberine hydrochloride (**Figure 5D**; **Supplementary Table S1**). These results indicated that Berberine hydrochloride mediated-pathogen resistance depended on the intestinal activation of PMK-1. To further confirm whether Berberine hydrochloride relies on intestinal PMK-1 to regulate innate immunity, we utilized AY102 transgenic worms, which express *pmk-1* under the intestinal *vha-6* promoter in a *pmk-1(km25)* mutant background. Expression of *pmk-1* in the intestine fully restored the survival rate of *pmk-1(km25)* mutants (**Figure 5E**; **Supplementary Table S1**). These results demonstrate that Berberine hydrochloride-induced innate immunity requires the intestinal activity of PMK-1.

### Berberine hydrochloride promotes innate immunity in mice via p38 MAPK pathway

**Figure 6:**
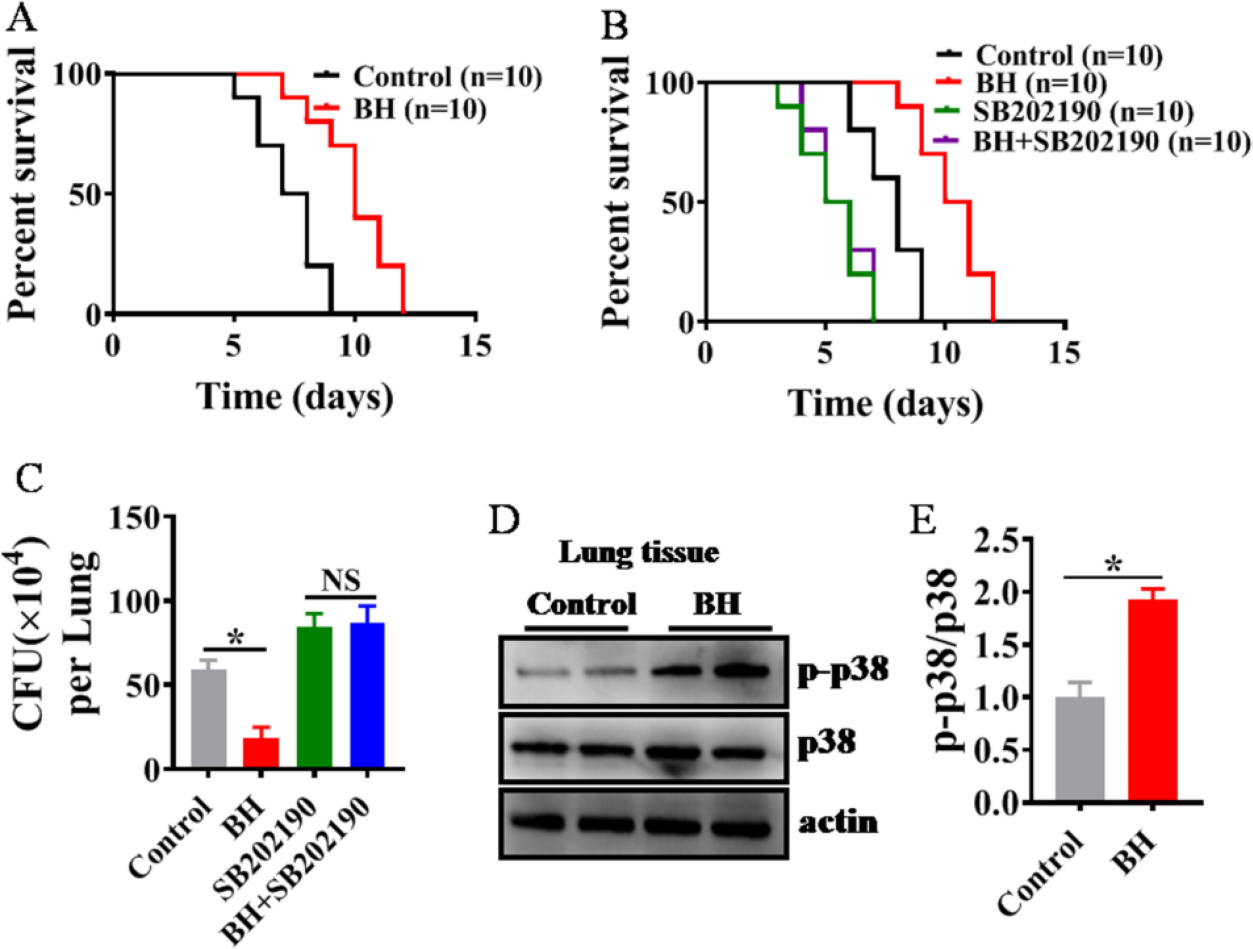
Berberine hydrochloride promotes innate immunity in mice via p38 MAPK pathway. **(A)** BH (10 mg/kg body weight) treated mice increases the resistance to *P. aeruginosa* PA14 infection compared with control mice. (n = 10). *P < 0.05 (log-rank test). **(B)** The p38 inhibitor SB202190 increased the susceptibility to *P. aeruginosa* PA14 infection compared with control mice *P < 0.05 (log-rank test) and suppressed the enhanced resistance to *P. aeruginosa* PA14 upon BH (10 mg/kg body weight) treatment. (n = 10). (log-rank test). **(C)** *P. aeruginosa* counts from lung homogenates plated on LB agar plates. (n = 6). **(D)** BH (10 mg/kg body weight) significantly increased the levels of active p38 in the lung. (n = 6). **(E)** The right panel shows quantification of phosphorylated p38 MAPK levels. These results are mean ± SEM of three independent experiments performed in triplicate. *P < 0.05 versus control (one-way ANOVA followed by a Student-Newman-Keuls test).

To investigate whether Berberine hydrochloride protects mice against *P. aeruginosa* infection, mice were treated with 10 mg/kg body weight of Berberine hydrochloride (29), while mice were infection with *P. aeruginosa* PA14 (1.0 × 10^6^ CFUs/mouse). Berberine hydrochloride-treated mice exhibited enhanced resistance to *P. aeruginosa* PA14 infection compared to control mice (**Figure 6A**). Since, the p38 MAPK pathway, conserved from worms to mammals (8), was assessed to determine if it mediates the Berberine hydrochloride-induced immune response. Mice were treated with 10 mg/kg Berberine hydrochloride (29) or 20 μg/kg of the p38 inhibitor SB202190 (8, 30), followed by *P. aeruginosa* PA14 infection (1.0 × 10^6^ CFUs/mouse). SB202190-treated mice showed increased susceptibility to infection compared to controls, and the inhibitor blocked the enhanced resistance induced by Berberine hydrochloride (**Figure 6B**). Bacterial loads in the lungs were quantified by colony-forming units (CFUs). Berberine hydrochloride-treated mice displayed significantly fewer CFUs of *P. aeruginosa* compared to controls (**Figure 6C**), whereas SB202190-treated mice had increased bacterial loads. However, the bacterial reduction conferred by Berberine hydrochloride was not observed when SB202190 was administered (**Figure 6C**). Additionally, Berberine hydrochloride significantly increased active PMK-1 levels in mice (**Figures 6D, E**). Collectively, these results suggest that Berberine hydrochloride enhances innate immunity in mice via the p38 MAPK pathway.

## Discussion

Berberine hydrochloride, a natural alkaloid derived from *Rhizomacoptidis*, has a long-standing history of use in Ayurvedic and traditional Chinese medicine, demonstrating diverse properties such as anti-cancer (3), antibacterial (4), anti-inflammatory (5) and anti-neurodegenerative (6) properties. However, its molecular mechanisms in enhancing innate immunity remain poorly understood. In this study, we reveal that preemptive treatment with Berberine hydrochloride protects *C. elegans* from Gram-negative pathogens like *Pseudomonas aeruginosa* and *Salmonella enterica*, as well as the Gram-positive pathogen *Listeria monocytogenes*. Notably, this protective effect involves a conserved innate immunity mechanism in *C. elegans*, mediated through the p38 MAPK pathway. Pathogen avoidance is a known component of the *C. elegans* defense mechanism against *Pseudomonas aeruginosa* (23). Therefore, we speculated whether Berberine hydrochloride promoted innate immunity by influencing the pathogen-avoidance behavior of *C. elegans* and found that 10 μM berberine hydrochloride had no effect on the pathogen avoidance behavior of *C. elegans* (**Figure 1D**). Our findings further demonstrate that the intestinal PMK-1 is essential for Berberine hydrochloride-induced enhancement of innate immunity, which reduces bacterial burden and shields worms from pathogen infection. At the same time, we also explored whether berberine hydrochloride could safeguard mice against *Pseudomonas aeruginosa* infection. The results showed that compared with the control group, berberine hydrochloride-treated mice showed increased resistance to PA14 infection (**Figure 6A**). To determine whether berberine hydrochloride enhances innate immunity in mice through the p38 MAPK pathway, we treated mice with the p38 inhibitor SB202190. We found that SB202190-treated mice had increased susceptibility to infection compared with the control group, and the inhibitor blocked the increase in resistance induced by berberine hydrochloride (**Figure 6B**). These results suggest that berberine hydrochloride enhances innate immunity in mice through the p38 MAPK pathway and the mechanism is conserved from worms to mammals. Overall, this study identifies an alternative mechanism, akin to antibiotic action, through which Berberine hydrochloride supports host defense against bacterial infections. This may provide new strategies for tackling drug-resistant infections.

Growing evidence suggests that natural products are emerging as promising agents for combating pathogens by modulating specific signaling pathways. For instance, the natural compound sanguinarine enhances healthspan and strengthens innate immunity in *C. elegans* through activation of the PMK-1/SKN-1-dependent pathway (19). Similarly, Brevilin A boosts innate immunity, extends lifespan, and improves resistance to oxidative stress in *C. elegans* via the p38 MAPK pathway (2). Luteolin has been shown to promote pathogen resistance in *C. elegans* through the DAF-2/DAF-16 insulin-like signaling pathway (31). While dioscin activates the endoplasmic reticulum unfolded protein response (UPR) to defend against pathogenic bacteria via the IRE-1/XBP-1 pathway (26). Additionally, Schisandrin A enhances innate immunity in both worms and mice by targeting the p38 MAPK pathway (32). These findings highlight the potential of leveraging immunomodulatory properties to develop novel anti-infective therapies (8). In this study, we demonstrate that Berberine hydrochloride activates the p38 MAPK pathway to enhance innate immunity in *C. elegans* and mice. Inflammation, a double-edged sword, can be detrimental when excessive but beneficial when moderate, aiding in the host’s defense against pathogens. In inflammatory animal models, BH may suppress the expression of p38-dependent pro-inflammatory cytokines, exerting an anti-inflammatory effect (33). Conversely, during pathogen infection, BH enhances innate immunity by increasing the expression of pro-inflammatory cytokines, thereby protecting the host (34). The evolutionary conservation of the p38 MAPK pathway underscores the potential of Berberine hydrochloride as a therapeutic candidate for treating human infectious diseases.

Currently, infectious diseases are one of the most formidable threats and impose a huge burden on the human medical system, and bacterial infections are one of the main causes of infectious diseases (35). Previous study has shown that natural products, serving as a crucial source of potential antimicrobial lead compounds, plays a significant role in the treatment of diseases, particularly infectious diseases (36). In the research on innate immunity, multiple model organisms have been employed as pathogen-animal models, including flies (37), mice (38), zebrafish (39), and *C. elegans* (40). Therefore, the role of Berberine hydrochloride in innate immunity and its potential molecular mechanisms were investigated by using *C. elegans*. However, *C. elegans* has a simple physiological structure, lacks an adaptive immune system, and shows significant differences in gene expression regulation and metabolic pathways compared with higher organisms, which presents certain limitations. In our study, we used *C. elegans* to identify that Berberine hydrochloride can enhance the host’s resistance to pathogenic bacteria. We then used a mouse model to further explore the role of berberine hydrochloride in promoting innate immunity in mice through the p38 MAPK pathway. Overall, using mouse and *C. elegans* models, we demonstrated that berberine hydrochloride enhanced innate immunity in mice through the p38 MAPK pathway and the mechanism was conserved from worms to mammals. At present, the mechanisms by which drugs inhibit gram-positive bacteria mainly include inhibiting the synthesis of the bacterial cell wall, interfering with the synthesis of bacterial proteins, inhibiting the synthesis of bacterial nucleic acids, and affecting the function of the bacterial cell membrane. It may be involved in the autophagy signaling pathway, the two- component signal transduction system (TCS), and the protein-synthesis-related signaling pathway, etc. Our study found that berberine can reduce bacterial accumulation in the host. This finding led us to wonder whether berberine affects the gut microbial community to maintain host health and resist pathogenic infections, or whether it affects metabolic pathways in host cells, such as energy metabolism and lipid metabolism. It is hoped that our study can provide a theoretical basis for the further development and utilization of berberine as a novel anti-infection agent or an intervention in immune-related diseases. It is hoped that through drug intervention, the immune system can be regulated, which will provide a theoretical basis for the further development and utilization of berberine hydrochloride as a new anti-infection or immune-related disease intervention drug.

## Materials and methods

### Chemicals

Berberine hydrochloride was obtained from Sigma Chemical Co. (St. Louis, MO) and dissolved in Dimethyl sulfoxide (DMSO) as a stock solution at a 100 mM concentration and stored in aliquots at -20°C. The experimental groups with different molar concentrations (5, 10, and 20 μM) were obtained by adding 5 μL, 10 μL, and 20 μL of 100 mM Berberine hydrochloride to 100 mL of Nematode Growth Medium (NGM). During this procedure, the concentration of DMSO in the medium ends up being much less than 0.02%. This will ensure the accuracy of the experimental results during the experiment.

### Strains

N2 Bristol wild-type, AU3 *nsy-1(ag3)*, AU1 *sek-1(ag1)*, KU25 *pmk-1(km25)*, CB1370 *daf-2(e1370)*, SD184 *mpk-1(n2521)*, YT17 *crh-1(tz2)*, CL691 *skn-1(zu67)*, VC8 *jnk-1(gk7)*, RB754 *aak-2(ok524)*, AU133 (*irg-1::GFP*), AU78 (*T24B8.5p::GFP*), SAL144 (*K08D8.5p::GFP*), AY102 *[pmk-1(km25);acEx102]*, NR222 *[rde-1(ne219);kzIs9]*, NR350 *[rde-1(ne219);kzIs20]*, VP303 *[rde-1(ne219) V; kbIs7]*, TU3401 *[sid-1(pk3321)V;uIs69V]* were obtained from the Caenorhabditis Genetics Center (CGC at the University of Minnesota, USA).

### RNAi experiment

The *E. coli* strain HT115(DE3) expressing dsRNA and the bacteria strain HT115 (DE3) containing the empty vector L4440 as a control were each added to LB liquid medium containing 100 μg/mL ampicillin, overnight culture at 37 ℃. The amplified bacterial solution was added to the NGM culture plate containing IPTG, dried and placed in a constant temperature incubator at 25 ℃ for 12 h. Then the synchroized larvae growing to L1 stage were added to the culture plate. These nematodes were cultured at 20 ℃ until L4 stage, and then verified by gene knock-down experiment, or used for later RNAi nematode pathogen infection experiment.

The strains of *E.coli* used for RNAi were obtained from the Ahringer library (41). *Unc-22* RNAi was included as a positive control in all experiments to account for RNAi efficiency.

### Infection assay

*Escherichia coli* OP50, *Listeria monocytogenes*, *Salmonella enterica* SL1344, and *Pseudomonas aeruginosa* PA14 were grown overnight in LB broth at 37°C, and took 100 μL of bacterial solution and spread to NGM plates. Then the NGM plate coated with the bacterial solution is placed in a biosafety cabinet to let the bacterial solution dry. The culture plate was incubated at 37 ℃ for 12 h to activate the virulence factors of *Listeria monocytogenes*, *Salmonella enterica* SL1344, and *Pseudomonas aeruginosa* PA14. All infection assay were performed on NGM agar plates or NGM plates supplemented with or without Berberine hydrochloride (0, 5, 10, 20 μM). The number of living worms were counted at 12 h intervals. Immobile adult worms unresponsive to touch were scored as dead (19, 42). Animals that climbed down from plates or showed unnatural deaths were censored. Unnatural death mainly refers to the death of nematodes caused by external factors or experimental operations. For example, improper use of worm pickers can cause mechanical damage to the worm bodies. Uneven temperature can cause nematodes to drill into the NGM plate or lead to their loss. All experiments were performed three times independently.

### Bacterial proliferation assay

Liquid bacterial growth was performed in 96-well microtiter plates containing the different bacterial strains as previously described (31, 43). The absorbance (OD 600 nm) was measured every 5 h for an 36 h incubation period with regular shaking at 37°C, 180 rpm. All experiments were performed three times independently.

### Fluorescence microscopy

Nematodes were synchronized and treated for 1 day with or without 10 μM Berberine hydrochloride starting at L4 larvae stage. The images were obtained by using Zeiss Axioskop 2 plus fluorescence microscope. Fluorescence intensity was quantified by using the ImageJ software (NIH). All experiments were performed three times independently.

### Quantitative real-time PCR

Nematodes were synchronized and treated for 1 day with or without 10 μM Berberine hydrochloride starting at L4 larvae stage. Total RNA was extracted from worms with TRIzolReagent (Invitrogen) as previously described (8). Using *pmp-3* for an internal controlas previously described (8). All experiments were performed three times independently. The following primers were used in this study:

*pmp-3* primers:

*pmp-3*-F: TGGATTGTCATTGGCGTCG.
*pmp-3*-R: GTTGTCGCAGAGTGGTGTTT.

*K08D8.5* primers:

*K08D8.5*-F: TGCTCGTCGGCTTCTCAAT.
*K08D8.5*-R: GCCGCAGTATCCAATCCCAT.

*lys-2* primers:

*lys-2*-F: CAAACAATCCAACTGCCAACG.
*lys-2*-R: TGGCTTTGTCTCTCCAGAAGT.

*F35E12.5* primers:

*F35E12.5*-F: TGGACCTGATTACACCGCTT.
*F35E12.5*-R: TGGAAATGAACAGCGAATCGG.

### Quantification of intestinal bacterial loads

Synchronized worms were cultivated on *E.coli* OP50 at 20°C until L4 larvae stage. Then, worms were transferred to NGM agar plates (supplemented with or without 10 μM Berberine hydrochloride) containing *P. aeruginosa*/GFP, *S. enterica*/GFP, or *L. monocytogenes*/GFP for 48h at 25°C (2, 23). The images were obtained by using a Zeiss Axioskop 2 plus fluorescence microscope. Fluorescence intensity was quantified by using the ImageJ software (NIH). All experiments were performed three times independently.

### Western blotting

Worms or lung tissue were homogenized in liquid nitrogen. Then, the homogenate was lysed on ice for 30 minutes in lysis buffer. 1. The lysates of total protein were loaded (60μg per well) and separated on a 10% SDS polyacrylamide gel. The preparation method refers to the instruction manual of the SDS-PAGE gel preparation kit. 2. Electrotransfer was performed after electrophoresis. A PVDF membrane with a diameter of 0.22 μm was soaked in methanol until the membrane became translucent. Then, the PVDF membrane was soaked in the electrotransfer solution. A membrane sandwich was made by placing the gel on it and covering it with the PVDF membrane. Electrotransfer was carried out at 300 mA for 90 min. 3. After being blocked with 5% skim milk powder for 2 h, the PVDF membrane was washed three times with TBST. 4. Phosphorylated PMK-1 protein was detected by using anti-active p38 polyclonal antibody from rabbit (Abcam, ab4822, 1:1000 dilution), and anti-beta actin antibodies (Abcam, ab227387,1:1000 dilution). Band intensities were measured using ImageJ software. All experiments were performed three times independently.

### Animal studies

C57BL/6mice were inoculated with *P. aeruginosa*-laden agarose beads, as previously described (8, 44). The average 50 µl agar-beads suspension contained 1.0 × 10^6^ CFUs/mouse. The agar-beads suspension was ready for inoculation in the lungs of mice by an intratracheal injection. Animals received daily doses of 10 mg/kg body weight Berberine hydrochloride and SB202190 (20 μg/kg/d) through intraperitoneal injection for 7 d. Each group had 10 animals.

ARRIVE guidelines Compliance: All methods are reported in accordance with ARRIVE guidelines.

### Statistics

Data were presented as mean ± SEM. Graphs were generated with GraphPad Prism 7.0 software (GraphPad, San Diego, CA, USA). Statistical analyses for all data were carried out using Student’s t-test (unpaired, two-tailed) or ANOVA after testing for equal distribution of the data and equal variances within the data set. Survival data were analyzed by using the log-rank (Mantel-Cox) test. All experiments were performed three times independently. *P*< 0.05 was considered significant.

## Data Availability

The original contributions presented in the study are included in the article/**Supplementary Material**. Further inquiries can be directed to the corresponding authors.
