# Supplementary material for: Berberine hydrochloride enhances innate immunity to protect against pathogen infection via p38 MAPK pathway
Source: Front Immunol. 2025 Feb 28;16:1536143. doi: 10.3389/fimmu.2025.1536143 (PMC11906452; doi:10.3389/fimmu.2025.1536143)

**Table S1**

| **Strain/Treatment** | **Replicate** | **Mean survival ± SEM (hours)** | **Censored/Total** | **Max survival ± SEM (hours)** | ***P* value** |
| --- | --- | --- | --- | --- | --- |
| **Figure 1A** |  |  |  |  |  |
| **Control** | **1** | **89.87±3.24** | **8/71** | **109.34±2.45** |  |
| **5 μM BH** |  | **90.48±2.58** | **9/75** | **110.94±3.56** | ***P* =0.277** |
| **10 μM BH** |  | **112.67±2.34** | **7/66** | **159.37±4.54** | ***P*＜0.05** |
| **20 μM BH** |  | **97.67±4.56** | **12/89** | **132.53±2.38** | ***P*＜0.05** |
| **Control** | **2** | **89.45±2.97** | **13/99** | **110.24±3.21** |  |
| **5 μM BH** |  | **90.64±3.64** | **12/93** | **110.67±3.52** | ***P* =0.203** |
| **10 μM BH** |  | **113.98±3.67** | **6/75** | **159.78±2.79** | ***P*＜0.05** |
| **20 μM BH** |  | **97.72±4.02** | **8/96** | **132.01±3.71** | ***P*＜0.05** |
| **Control** | **3** | **89.43±2.65** | **9/94** | **109.02±4.68** |  |
| **5 μM BH** |  | **89.93±2.94** | **10/98** | **110.81±2.59** | ***P* =0.191** |
| **10 μM BH** |  | **112.45±3.64** | **17/103** | **159.96±2.82** | ***P*＜0.05** |
| **20 μM BH** |  | **98.92±2.51** | **8/95** | **131.98±3.46** | ***P*＜0.05** |
| **Figure 2A** |  |  |  |  |  |
| ***Salmonella enterica*** | |  |  |  |  |
| **Control** | **1** | **194.42±2.41** | **9/83** | **266.51±3.42** |  |
| **BH** |  | **263.05±3.79** | **6/76** | **335.63±4.38** | ***P*＜0.05** |
| **Control** | **2** | **194.28±2.98** | **11/88** | **266.01±2.73** |  |
| **BH** |  | **264.97±2.64** | **10/90** | **336.54±3.19** | ***P*＜0.05** |
| **Control** | **3** | **194.98±3.06** | **6/79** | **265.95±3.52** |  |
| **BH** |  | **263.69±3.13** | **9/72** | **334.91±3.64** | ***P*＜0.05** |
| **Figure 2 B** |  |  |  |  |  |
| ***Listeria monocytogenes*** | |  |  |  |  |
| **Control** | **1** | **89.25±3.87** | **14/125** | **108.38±3.73** |  |
| **BH** |  | **107.45±2.86** | **10/94** | **152.49±4.61** | ***P*＜0.05** |
| **Control** | **2** | **88.56±3.74** | **12/118** | **109.51±2.74** |  |
| **BH** |  | **108.63±2.67** | **18/129** | **153.93±2.81** | ***P*＜0.05** |
| **Control** | **3** | **88.94±2.95** | **15/116** | **108.92±2.64** |  |
| **BH** |  | **108.69±3.51** | **17/121** | **152.46±3.49** | ***P*＜0.05** |
| **Figure 3A** |  |  |  |  |  |
| **N2** | **1** | **88.87±3.14** | **9/87** | **108.24±2.85** |  |
| **N2+ BH** |  | **113.67±2.67** | **11/93** | **156.17±2.14** | ***P*＜0.05** |
| **N2** | **2** | **89.26±3.12** | **7/78** | **109.37±2.48** |  |
| **N2+ BH** |  | **112.38±2.02** | **8/80** | **156.43±2.75** | ***P*＜0.05** |
| **N2** | **3** | **88.52±2.47** | **10/95** | **109.19±3.59** |  |
| **N2+ BH** |  | **112.65±3.59** | **11/83** | **157.58±3.66** | ***P*＜0.05** |
| **Figure 3B** |  |  |  |  |  |
| ***pmk-1(km25)*** | **1** | **49.23±2.49** | **10/84** | **79.49±2.47** |  |
| ***pmk-1(km25)* + BH** |  | **49.47±2.85** | **13/89** | **78.22±3.62** | ***P* =0.232** |
| ***pmk-1(km25)*** | **2** | **48.94±2.54** | **9/98** | **79.46±3.35** |  |
| ***pmk-1(km25)* + BH** |  | **48.53±2.38** | **7/82** | **79.25±2.74** | ***P* =0.304** |
| ***pmk-1(km25)*** | **3** | **49.46±2.84** | **9/85** | **78.31±2.84** |  |
| ***pmk-1(km25)* + BH** |  | **48.19±2.46** | **11/97** | **79.94±3.85** | ***P* =0.248** |
| **Figure 3C** |  |  |  |  |  |
| ***mpk-1(n2521)*** | **1** | **72.57±3.15** | **7/65** | **84.16±2.15** |  |
| ***mpk-1(n2521)*＋ BH** |  | **96.34±2.36** | **6/71** | **108.18±2.64** | ***P*＜0.05** |
| ***mpk-1(n2521)*** | **2** | **72.68±3.52** | **8/69** | **84.35±2.45** |  |
| ***mpk-1(n2521)*＋ BH** |  | **96.39±2.12** | **7/62** | **108.45±2.15** | ***P*＜0.05** |
| ***mpk-1(n2521)*** | **3** | **72.76±2.78** | **6/56** | **84.14±3.26** |  |
| ***mpk-1(n2521)*＋ BH** |  | **96.35±3.53** | **11/73** | **108.75±3.21** | ***P*＜0.05** |
| **Figure 3D** |  |  |  |  |  |
| ***daf-2(e1370)*** | **1** | **122.45±2.15** | **10/75** | **139.65±2.47** |  |
| ***daf-2(e1370)* + BH** |  | **142.65±2.12** | **7/73** | **168.27±3.82** | ***P*＜0.05** |
| ***daf-2(e1370)*** | **2** | **122.57±2.58** | **9/67** | **139.47±3.89** |  |
| ***daf-2(e1370)* + BH** |  | **142.69±2.23** | **5/68** | **169.41±2.45** | ***P*＜0.05** |
| ***daf-2(e1370)*** | **3** | **121.46±2.78** | **8/76** | **138.21±2.25** |  |
| ***daf-2(e1370)* + BH** |  | **143.22±2.54** | **8/63** | **169.84±3.14** | ***P*＜0.05** |
| **Figure 3E** |  |  |  |  |  |
| ***nsy-1(ag3)*** | **1** | **44.65±3.12** | **12/80** | **72.89±3.58** |  |
| ***nsy-1(ag3)* + BH** |  | **45.38±2.56** | **11/76** | **72.15±4.76** | ***P* =0.158** |
| ***nsy-1(ag3)*** | **2** | **44.23±3.15** | **7/72** | **72.42±2.52** |  |
| ***nsy-1(ag3)* + BH** |  | **45.54±1.68** | **5/64** | **72.45±3.34** | ***P* =0.252** |
| ***nsy-1(ag3)*** | **3** | **45.72±2.31** | **11/90** | **72.25±2.38** |  |
| ***nsy-1(ag3)* + BH** |  | **45.59±3.65** | **5/56** | **72.54±3.17** | ***P* =0.135** |
| **Figure 3F** |  |  |  |  |  |
| ***sek-1(ag1)*** | **1** | **44.12±3.34** | **8/77** | **72.34±3.27** |  |
| ***sek-1(ag1)* + BH** |  | **44.35±1.56** | **12/73** | **72.17±3.76** | ***P* =0.218** |
| ***sek-1(ag1)*** | **2** | **44.28±3.32** | **7/68** | **72.79±2.13** |  |
| ***sek-1(ag1)* + BH** |  | **45.14±1.65** | **9/82** | **72.35±3.67** | ***P* =0.263** |
| ***sek-1(ag1)*** | **3** | **45.82±2.45** | **11/75** | **72.26±2.37** |  |
| ***sek-1(ag1)* + BH** |  | **45.12±3.16** | **9/69** | **72.23±3.15** | ***P* =0.174** |
| **Figure 5A TU3401** |  |  |  |  |  |
| **EV** | **1** | **89.85±3.47** | **12/83** | **107.36±3.55** |  |
| **EV+BH** |  | **113.36±2.86** | **12/85** | **146.65±4.26** | ***P*＜0.05** |
| ***pmk-1* RNAi** |  | **88.58±3.02** | **6/72** | **108.02±2.42** |  |
| ***pmk-1* RNAi+BH** |  | **112.54±4.75** | **9/88** | **145.85±3.14** | ***P*＜0.05** |
| **EV** | **2** | **88.75±2.39** | **7/53** | **108.74±2.36** |  |
| **EV+BH** |  | **112.69±3.67** | **8/79** | **146.84±3.11** | ***P*＜0.05** |
| ***pmk-1* RNAi** |  | **88.48±3.75** | **9/86** | **108.26±2.48** |  |
| ***pmk-1* RNAi+BH** |  | **111.96±3.37** | **7/83** | **146.21±2.36** | ***P*＜0.05** |
| **EV** | **3** | **88.69±2.57** | **12/91** | **107.63±3.58** |  |
| **EV+BH** |  | **112.57±2.48** | **9/78** | **146.79±3.04** | ***P*＜0.05** |
| ***pmk-1* RNAi** |  | **89.94±3.86** | **10/86** | **108.98±2.42** |  |
| ***pmk-1* RNAi+BH** |  | **113.48±3.21** | **10/89** | **145.37±2.56** | ***P*＜0.05** |
| **Figure 5B NR350** |  |  |  |  |  |
| **EV** | **1** | **88.37±2.45** | **9/79** | **108.73±3.24** |  |
| **EV+BH** |  | **113.35±4.59** | **8/90** | **146.56±3.74** | ***P*＜0.05** |
| ***pmk-1* RNAi** |  | **88.61±2.68** | **6/59** | **107.78±2.49** |  |
| ***pmk-1* RNAi+BH** |  | **114.95±2.55** | **9/64** | **145.42±3.37** | ***P*＜0.05** |
| **EV** | **2** | **89.53±3.57** | **10/74** | **107.57±2.44** |  |
| **EV+BH** |  | **113.51±2.19** | **10/68** | **145.56±4.74** | ***P*＜0.05** |
| ***pmk-1* RNAi** |  | **88.98±3.65** | **5/62** | **108.49±3.21** |  |
| ***pmk-1* RNAi+BH** |  | **114.37±2.89** | **9/59** | **146.16±3.64** | ***P*＜0.05** |
| **EV** | **3** | **88.13±2.32** | **10/63** | **107.25±3.62** |  |
| **EV+BH** |  | **114.79±4.67** | **9/74** | **145.59±2.83** | ***P*＜0.05** |
| ***pmk-1* RNAi** |  | **88.46±3.84** | **9/62** | **107.39±3.01** |  |
| ***pmk-1* RNAi+BH** |  | **113.79±2.39** | **8/61** | **146.42±4.47** | ***P*＜0.05** |
| **Figure 5C NR222** |  |  |  |  |  |
| **EV** | **1** | **89.64±2.45** | **17/115** | **108.65±2.75** |  |
| **EV+BH** |  | **112.47±4.69** | **9/89** | **144.34±4.96** | ***P*＜0.05** |
| ***pmk-1* RNAi** |  | **88.59±3.37** | **8/82** | **107.24±2.44** |  |
| ***pmk-1* RNAi+BH** |  | **112.95±2.74** | **10/99** | **143.45±2.57** | ***P*＜0.05** |
| **EV** | **2** | **89.47±3.97** | **19/126** | **108.85±3.05** |  |
| **EV+BH** |  | **112.74±3.21** | **11/109** | **144.75±2.48** | ***P*＜0.05** |
| ***pmk-1* RNAi** |  | **89.97±2.64** | **9/84** | **107.58±3.67** |  |
| ***pmk-1* RNAi+BH** |  | **112.85±2.98** | **12/90** | **143.92±4.45** | ***P*＜0.05** |
| **EV** | **3** | **88.62±4.26** | **15/105** | **108.59±3.56** |  |
| **EV+BH** |  | **113.84±2.86** | **13/101** | **144.46±2.28** | ***P*＜0.05** |
| ***pmk-1* RNAi** |  | **88.39±2.59** | **9/95** | **108.27±3.41** |  |
| ***pmk-1* RNAi+BH** |  | **112.68±4.74** | **10/85** | **144.15±3.96** | ***P*＜0.05** |
| **Figure 5D VP303** |  |  |  |  |  |
| **EV** | **1** | **89.37±2.37** | **10/80** | **109.67±2.43** |  |
| **EV+BH** |  | **114.48±2.82** | **6/74** | **143.68±4.56** | ***P*＜0.05** |
| ***pmk-1* RNAi** |  | **50.86±3.58** | **9/77** | **85.36±3.43** |  |
| ***pmk-1* RNAi+BH** |  | **49.56±3.91** | **8/73** | **86.53±2.47** | ***P* =0.235** |
| **EV** | **2** | **88.93±2.46** | **9/80** | **109.47±3.95** |  |
| **EV+BH** |  | **115.87±4.44** | **8/83** | **143.46±2.37** | ***P*＜0.05** |
| ***pmk-1* RNAi** |  | **50.15±3.86** | **5/74** | **86.75±3.28** |  |
| ***pmk-1* RNAi+BH** |  | **50.25±3.45** | **6/68** | **85.86±2.37** | ***P* =0.303** |
| **EV** | **3** | **88.78±2.42** | **12/87** | **108.81±2.61** |  |
| **EV+BH** |  | **114.59±2.49** | **10/87** | **144.86±3.27** | ***P*＜0.05** |
| ***pmk-1* RNAi** |  | **50.92±3.29** | **13/94** | **86.74±2.17** |  |
| ***pmk-1* RNAi+BH** |  | **49.36±4.36** | **8/66** | **85.53±3.63** | ***P* =0.258** |
| **Figure 5E** |  |  |  |  |  |
| **N2** | **1** | **89.65±3.28** | **11/70** | **108.47±2.85** |  |
| **N2+BH** |  | **116.62±3.23** | **11/82** | **146.98±4.38** | ***P*＜0.05** |
| ***pmk-1(km25)*** |  | **49.58±4.53** | **8/58** | **78.86±2.46** |  |
| ***pmk-1(km25)*+BH** |  | **49.63±3.64** | **10/79** | **78.67±3.25** | ***P* =0.245** |
| **AY102** |  | **88.77±4.48** | **8/64** | **109.85±2.29** |  |
| **AY102+BH** |  | **115.79±2.35** | **9/73** | **146.69±4.63** | ***P*＜0.05** |
| **N2** | **2** | **89.63±3.68** | **9/61** | **108.83±2.52** |  |
| **N2+BH** |  | **116.36±2.74** | **11/91** | **147.83±2.64** | ***P*＜0.05** |
| ***pmk-1(km25)*** |  | **48.71±3.63** | **7/69** | **79.87±2.43** |  |
| ***pmk-1(km25)*+BH** |  | **48.48±2.96** | **10/73** | **78.93±1.94** | ***P* =0.231** |
| **AY102** |  | **88.85±4.42** | **5/66** | **108.51±3.07** |  |
| **AY102+BH** |  | **116.73±3.67** | **9/71** | **146.24±3.36** | ***P*＜0.05** |
| **N2** | **3** | **89.73±2.63** | **13/97** | **108.12±2.23** |  |
| **N2+BH** |  | **117.85±2.29** | **12/99** | **147.67±3.56** | ***P*＜0.05** |
| ***pmk-1(km25)*** |  | **48.69±3.15** | **5/59** | **78.53±4.29** |  |
| ***pmk-1(km25)*+BH** |  | **49.68±4.53** | **4/63** | **79.67±3.64** | ***P* =0.214** |
| **AY102** |  | **88.64±2.83** | **7/64** | **108.79±2.91** |  |
| **AY102+BH** |  | **116.37±2.39** | **8/65** | **147.48±2.89** | ***P*＜0.05** |

**Figure S1** BH enhanced resistance to *P. aeruginosa* PA14 in WT (N2) (A), *crh-1(tz2)* (B), *aak-2(ok524)* (C), *jnk-1(gk7)* (D). (log-rank test). (n＞ 40).


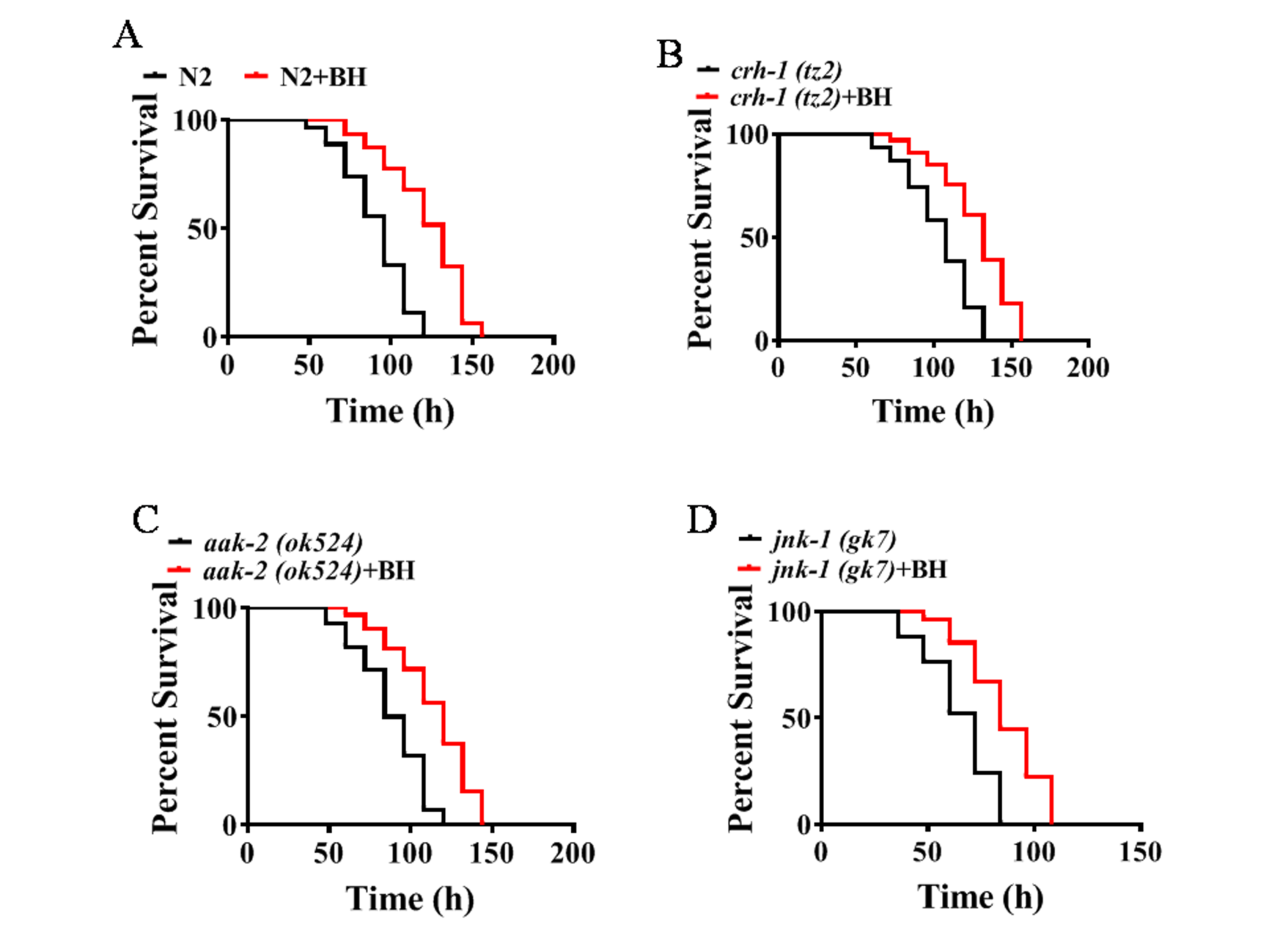

Supplement: Supplementary file 1 [file DataSheet1.docx]
